# Symptomatic familial adenomatous polyposis in an adolescent: A case report

**DOI:** 10.1016/j.ijscr.2021.106118

**Published:** 2021-06-26

**Authors:** Dinesh Prasad Koirala, Bibek Man Shrestha, Suraj Shrestha, Suraj Bhatta, Sanjeev Kharel, Sansar Babu Tiwari, Vivek Karn, Om Prakash Bhatta

**Affiliations:** a Department of GI and General Surgery, Tribhuvan University Teaching Hospital, Institute of Medicine, Kathmandu, Nepal; b Maharajgunj Medical Campus, Institute of Medicine, Kathmandu, Nepal; c Department of Pathology, Tribhuvan University Teaching Hospital, Institute of Medicine, Kathmandu, Nepal

**Keywords:** Familial adenomatous polyposis, Colonoscopy, Total proctocolectomy

## Abstract

**Introduction:**

Familial adenomatous polyposis (FAP) is an inherited colorectal cancer syndrome characterized by several adenomatous polyps of the gastrointestinal mucosa with a universal risk of colorectal cancer in a lifetime. FAP is usually asymptomatic in the first decade of life.

**Case presentation:**

We report a case of a 13-year-old girl diagnosed with FAP who presented in our center with symptoms of hematochezia along with a positive history of the untimely demise of her father and elder sister with similar symptoms.

**Discussion:**

FAP is an autosomal dominant disease affecting both male and female equally with variable penetrance. Diagnosis is made by finding hundreds to thousands of adenomatous polyps in the colon and rectum, and molecular analysis of the *APC* gene which forms the definitive diagnosis. Prophylactic laparoscopic total proctocolectomy with ileorectal anastomosis is a safe and feasible surgical option with a low risk of complications among adolescents. An endoscopic/colonoscopic procedure is recommended every 6 to 12 months after surgery to assess the anastomosis site, pouch, and residual rectum.

**Conclusion:**

FAP, a rare disease entity in adolescents should be managed by appropriate diagnostic procedures, early prophylactic surgery, and regular lifelong follow-up.

## Highlights

• FAP is rarely symptomatic in the first decade of life.
• Definitive diagnosis is based on molecular analysis of the APC gene.
• Early prophylactic surgery and regular surveillance is imperative.

## 1 Introduction

Familial adenomatous polyposis (FAP) is an inherited colorectal cancer syndrome characterized by several adenomatous polyps of the gastrointestinal (GI) mucosa which has a near 100% lifetime risk of colorectal cancer (CRC) with an incidence of approximately 1 in 7000 to 30,000 births [1,2].

Male and female offspring are at equal risk of inheriting the condition with no influence of the parental gender and the disease commonly has an onset of an average of 15 years [3]. Before this age, the disease is reported to be asymptomatic and with no macroscopically visible polyps [4].

Most commonly, FAP results from a germline pathogenic mutation in the adenomatous polyposis coli (*APC*) tumor suppressor gene on chromosome 5 [5]. The primary result of this mutation is the development of >100 colorectal adenomas almost universally [6]. The presentation is usually asymptomatic in the majority but may present with diarrhea, bleeding per rectum and abdominal pain, tenesmus, and obstruction, usually in the second to the third decade of life [7].

There are very few symptomatic cases of FAP in the first decade of life [6]. Cases of FAP are rarely reported from Nepal. Here, we report a case of a 13-year girl with symptoms of hematochezia who was subsequently diagnosed with FAP. This case has been reported in line with SCARE criteria [8].

## 2 Case presentation

A 13-year female presented to our center with bright red per rectal bleeding along with dull lower abdominal pain and occasional diarrhea for the past two years. She also complains of occasional fever. She denies headache, vomiting, loss of weight, or anorexia. There was a history of the untimely demise of her father at the age of 34 years and elder sister at the age of 14 years with a similar history of per rectal bleed but the entity was undiagnosed.

On examination, she was pale, afebrile, and hemodynamically stable. The abdomen was soft, non-tender, and non-distended. Examination of other systems including fundoscopy was unremarkable.

**Fig. 1:**
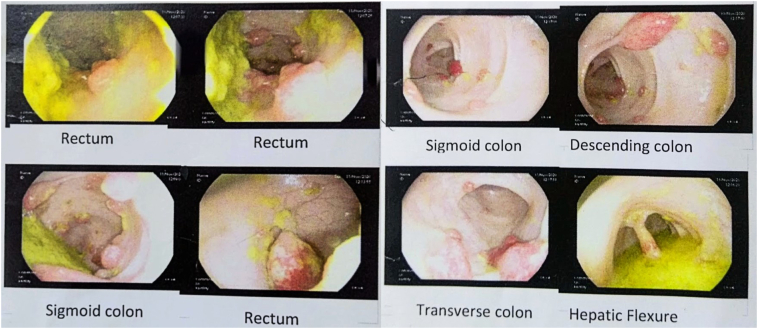
Colonoscopy showing multiple polyps in various parts of the colon and rectum.

**Fig. 2:**
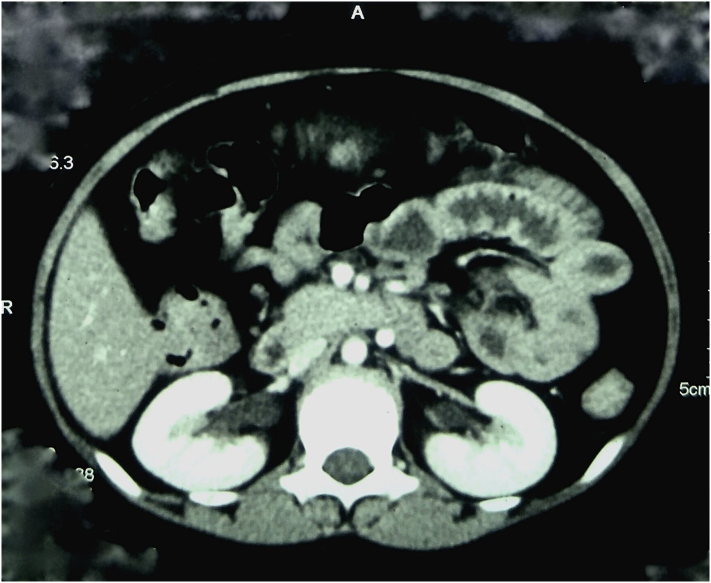
CT abdomen showing multiple heterogeneously enhancing polypoidal endophytic soft tissue in the rectum, sigmoid colon, descending colon, mid aspect of the transverse colon, and ascending colon.

Blood counts were within normal limits however the patient was anemic (Hemoglobin-8.2 g/dl, PCV-27 g %). All other blood investigations were within normal limits including tumor markers; CEA: 1.08 (<3 ng/ml) and CA-19.9: 6.9 (<37 U/ml). Colonoscopy revealed multiple pedunculated and sessile colonic polyps (>100) from rectum to cecum and the biopsy from the rectal polyp was consistent with focal active colitis (Fig. 1). CT scan of abdomen and pelvis revealed multiple heterogeneously enhancing polypoidal endophytic soft tissue density attached to bowel wall in the rectum, sigmoid colon, descending colon, mid aspect of the transverse colon, and ascending colon (Fig. 2).

**Fig. 3:**
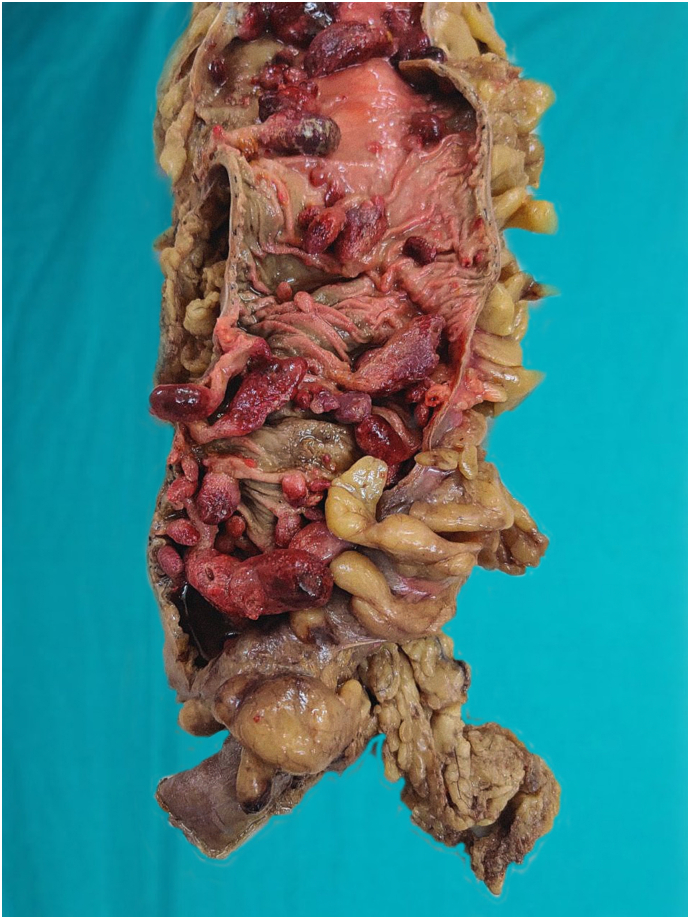
Ascending colon shows multiple pedunculated polyps, the largest one measuring 5 × 1.5 cm.

**Fig. 4:**
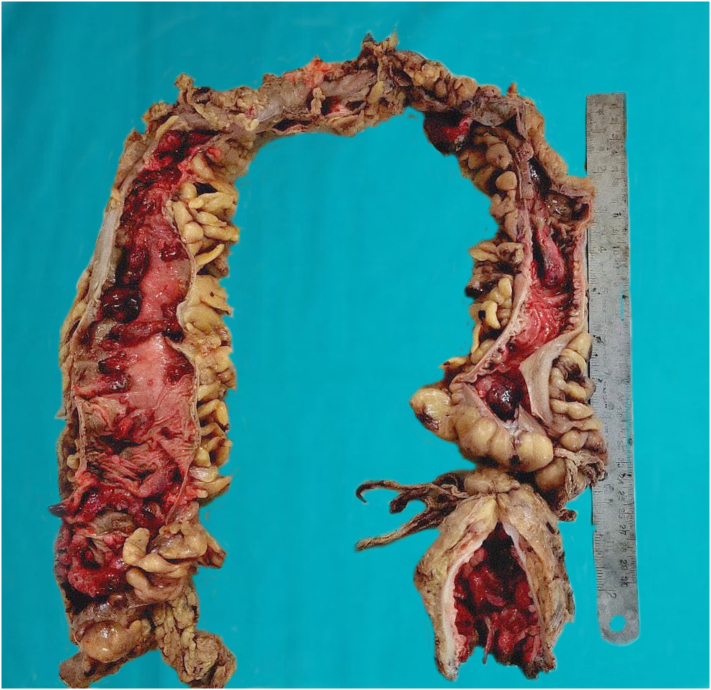
Total proctocolectomy specimen shows multiple pedunculated polyps throughout the length of the colon.

**Fig. 5:**
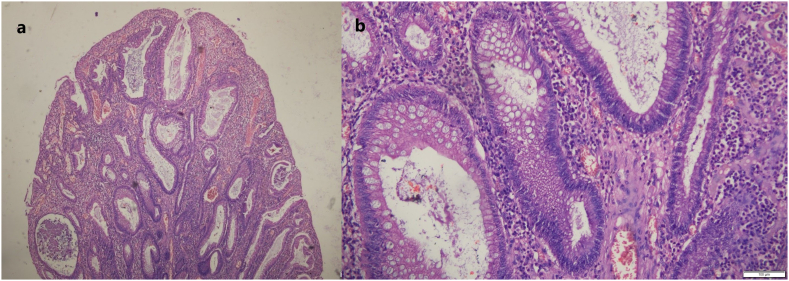
Section from the excised specimen shows a. multiple polyps and cystically dilated glands with edematous stroma b. Lining epithelium of the cyst shows focal dysplasia in the form of nuclear stratification and hyperchromatic nuclei.

After correction of the blood parameter with blood transfusion, the patient underwent laparoscopic total proctocolectomy in two settings. Initially, laparoscopically caecum, ascending colon, transverse colon, descending colon, sigmoid colon and rectum were mobilized ligating all vessels such as left colic and right colic artery. In the second phase, with lower midline incision, the mobilized large intestine with rectum were resected and ileoileal pouch was made. Soave procedure was used to pull through the ileal pouch with a subsequent loop ileostomy. Thus, ileal anal pouch anastomosis with loop ileostomy was performed by the experienced team of gastrointestinal surgeons of Tribhuvan University Teaching Hospital and was well tolerable by the patient. Gross examination of the resected specimen showed multiple pedunculated polyps (>500) all over the colon (Fig. 3, Fig. 4). Microscopic examination of the polyps showed cystically dilated glands with neutrophilic infiltrations into the lumen with focal dysplasia in the glands in the form of nuclear stratification, hyperchromatic nuclei, and loss of apical mucin (Fig. 5). Due to the unavailability of the needed screening tools in our facility and the financial constraints of the patient, genetic testing was not conducted in this patient and high-risk family members.

The post-operative period was uneventful and the patient recovered well. The patient is doing well with no complaints and remains disease-free at 6 months of follow-up. She is planned for a yearly follow-up.

## 3 Discussion

FAP is a high penetrance autosomal dominant disease affecting males and females equally [2]. A significant subset (approximately 20–30%) may arise from de novo mutations, though most affected individuals have a family history of FAP syndrome. [9]. Our patient had a similar illness in his father and elder sister but no manifestation of the disease in his other sibling to date.

CT imaging and colonoscopy play an important role in the diagnosis for the evaluation of hematochezia even in younger individuals especially with high-risk features such as anemia and significant family history [10]. As said, the diagnosis in our case was aided by a CT scan of the abdomen and colonoscopy for the evaluation of per rectal bleed, anemia, and positive family history.

The development of hundreds to thousands of adenomatous polyps in the colon and rectum, starting from childhood with an almost inevitable progression to CRC by the fourth decade of life is the hallmark of FAP [6]. The diagnosis in our case was made robust after the examination of the excised colon which revealed the presence of more than 500 polyps in the colon without any malignant changes in the adenomas. However, molecular analysis of *APC* is required for the definitive diagnosis of FAP [6]. Because clinically it is difficult to distinguish juvenile polyposis syndrome (JPS) especially with concomitant dysplasia and adenocarcinoma from FAP, JPS should also be considered in patients with multiple pedunculated polyps. For accurate diagnosis and differentiating FAP from JPS, sampling of more polyps for pathological examination and germline gene mutation tests are crucial along with other comprehensive evaluation of clinical presentation, endoscopic appearance, and genetic investigations and not merely based on presence or absence of adenomatous polyp [11]. Considering a poor financial background and a lack of genetic testing in our institution, genetic analysis was not performed in our case. However, the strong positive family history, presence of numerous polyps in the colon, and onset in the adolescent strongly suggest FAP in our case.

Annual screening for classic FAP by flexible sigmoidoscopy or colonoscopy typically begins at age of 10–12 years [12]. Thus, in patients affected by FAP, a screening colonoscopy with timely treatment of identified lesions has led to a 55% decrease in CRC as the first presenting sign leading to improvement in cumulative survival for the patients [13]. The family members of the patient were unaware of the condition and probable cause of death of their father and their elder daughter.

Surgery is the cornerstone in the management once FAP is diagnosed and the standard surgical options include total proctocolectomy (TC) with Brooke's ileostomy or ileal pouch with ileoanal anastomosis and subtotal colectomy with ileorectal anastomosis [14]. Cases in which colectomy is indicated include symptomatic polyps, advanced adenomas including CRC, severe or progressive polyposis, a polyp burden that cannot effectively be managed by endoscopy, or when surveillance is otherwise impossible [15]. Especially in young patients with a long life expectancy and with no advanced dysplasia and/or cancer, prophylactic surgery with ileorectal anastomosis (IRA) allowing preservation of the rectum should be considered in contributing to the quality of life without affecting the prognosis [16]. For prophylactic purposes, laparoscopic TC-IRA is safe and feasible in young patients with low risk of complications and with good outcomes [17]. Our patient underwent laparoscopic total proctocolectomy with ileal pouch anastomosis due to polyp even in the rectum along with a loop ileostomy.

The risk for upper gastrointestinal cancers and mesenteric desmoids in patients with FAP is present despite colectomy causing post-surgical morbidity or reduced lifespan [18]. For the assessment of anastomosis site, pouch, and residual rectum, regular endoscopic/colonoscopic procedure after surgery is recommended every 6–12 months [19]. In the case of the retained rectum, annual surveillance is vital [20]. Our patient is under regular follow-up and supervision.

In addition, individuals with first-degree relatives known to have FAP, patients with more than 10 to 20 intestinal polyps, and/or patients with colorectal adenomas in combination with extraintestinal manifestations associated with FAP are at high risk for FAP and testing should be discussed [15]. No family members in our case have undergone any genetic testing to date and also were reluctant to undergo any sort of genetic testing considering the financial status of the family.

## 4 Conclusion

Symptomatic FAP is rare in adolescents. Early interventions including prophylactic surgery, and regular surveillance is crucial for the prevention of lethal colorectal carcinoma and better quality of life without affecting the prognosis.

## Declaration of competing interest

None.
